# The effect of changing COVID-19 restrictions on the transmission rate in a veterinary clinic

**DOI:** 10.1016/j.idm.2023.01.005

**Published:** 2023-02-10

**Authors:** Lee Spence, David E. Anderson, Ibrahim Halil Aslan, Mahir Demir, Chika C. Okafor, Marcy Souza, Suzanne Lenhart

**Affiliations:** a Department of Mathematics, University of Tennessee, Knoxville, TN, USA; b Department of Large Animal Clinical Sciences, College of Veterinary Medicine, University of Tennessee, Knoxville, TN, USA; c Hopkins Marine Station, Stanford Doerr School of Sustainability, USA; d Department of Mathematics, Giresun University, Giresun, Turkey; e Department of Biomedical and Diagnostic Sciences, College of Veterinary Medicine, University of Tennessee, Knoxville, TN, USA

**Keywords:** Veterinary teaching hospital, COVID-19, Mathematical model, Disease transmission, 34A34, 92D30

## Abstract

With the declaration of the COVID-19 pandemic by the World Health Organization on March 11, 2020, the University of Tennessee College of Veterinary Medicine (UTCVM), like other institutions, restructured their services to reduce the potential spread of the COVID-19 virus while simultaneously providing critical and essential veterinary services. A mathematical model was developed to predict the change in the level of possible COVID-19 infections due to the increased number of potential contacts within the UTCVM hospital. A system of ordinary differential equations with different compartments for UTCVM individuals and the Knox county population was formulated to show the dynamics of transmission and the number of confirmed cases. Key transmission rates in the model were estimated using COVID-19 case data from the surrounding county and UTCVM personnel. Simulations from this model show the increasing number of COVID-19 cases among UTCVM personnel as the number of daily clients and the number of veterinary staff in the clinic are increased. We also investigate how changes within the Knox county community impact the UTCVM hospital. These scenarios show the importance of understanding the effects of re-opening scenarios in veterinary teaching hospitals.

## 1 Introduction

In December of 2019, reports of a novel, highly contagious coronavirus emerged, initially from China and a few weeks later identified in the USA in Washington State (Carvalho et al., 2021). The virus began to rapidly spread throughout the United States particularly in communities with high population density such as larger metropolitan areas (Fauver et al., 2020). Investigators initially estimated that this novel coronavirus, eventually named SARS-CoV-2 and then commonly referred to as COVID-19, had a basic reproductive number *R*_0_ likely exceeding two (Renardy et al., 2020; Xu et al., 2020; Zhuang et al., 2020). On March 11, 2020, the rapid global spread of the COVID-19 virus, led to the declaration of a pandemic by the World Health Organization (World Health Organization Director, 2020). Colleges and universities initiated emergency operations, but responses varied across institutions, ranging from closing to all in-person activities to maintaining some presence on-campus with varying degrees of in-person options. Many institutions closed campus at spring break and did not allow students to return after the holiday (Sahu, 2020).

Veterinary and human medical colleges are unique because they serve an essential role in human and animal health. These colleges have ethical and moral responsibilities that require continuous provision of health care to seriously ill patients. In addition, medical students require hands-on and in-person training to develop the skills and competencies needed to practice, as many medical services and training of clinical skills cannot be performed online. Hence, medical teaching hospitals rapidly developed protocols and biosafety procedures to ensure continuity of care for emergency and urgent care patients (Bhattacharjee et al., 2020; Gan et al., 2020; Sud et al., 2020). Elective procedures were canceled until more information could be obtained about how to manage the risk of COVID-19 exposure in a medical setting where close contact is common (Sahi et al., 2020).

Many mathematical models have included key features of the spread of the COVID-19 disease, such as distinguishing between asymptomatic infected individuals from the pre-symptomatic and symptomatic infected individuals (Chang et al., 2021; Keskinocak et al., 2020; Parajuli et al., 2020), while some early papers did not include asymptomatic compartments (Mukandavire et al., 2020; Nyabadza et al., 2020). Many different modeling techniques have been used, ranging from agent-based approaches to Bayesian modeling frameworks at the city level or county level (Murtadha et al.; Olmo & Sanso-Navarro, 2021; Potgieter et al., 2021). Some ODE models have included compartments with individuals in the hospital, with some assuming those persons to be isolated or others assuming them to be transmitting disease but at a lower rate (Edholm et al., 2022; Gumel et al., 2021; Ngonghala et al., 2020). Spatial scales in COVID-19 models have ranged from countries and counties to cities (Aslan et al., 2022; Gao et al., 2020; Gatto et al., 2020; Gumel et al., 2021; Macdonald et al., 2020; Renardy et al., 2020; Zhao & Feng, 2020). A few ODE models have represented COVID-19 transmission among health care workers in hospitals to illustrate spread and to consider interventions (Evans et al., 2021; Gozalpour et al., 2021). No ODE models have represented the spread from interactions between the public and staff at a veterinary hospital. Note that the clients spend a short time interacting with UTCVM staff, while the animals may stay longer in the clinic.

Although college biosafety procedures appeared to be effective, the need for novel predictive models to help assess risk and guide intervention strategies was urgent (Wang et al., 2020). Therefore, the objective of this study was to develop a predictive model that informs potential risk levels of intra-UTCVM infection that arise with increasing interactions with the Knox county public. We had to choose between two modeling approaches, Lagrangian and Eulerian, because our model involved two locations (in the veterinary clinic and in the county outside the clinic) and two groups of people (veterinary clinicians/staff and the county population, who do not work at the clinic) (Brauer et al., 2019; Cosner et al., 2009; Lee et al., 2020). Because the Eulerian approach typically represents migration from one patch to another, it was unsuitable for our situation. We chose a Lagrangian approach because the UTCVM clients remain county residents who visit the clinic for a short period of time each day (Bichara & Castillo-Chavez, 2016; Bichara et al., 2015). The outcomes of this model would provide science-based guidelines for relaxing the current restrictions at UTCVM and serving more patients and community health needs. Furthermore, it would provide a template that other veterinary colleges with similar structures to UTCVM could adapt in making decisions about their re-opening process.

In the next section, we formulate our model using a system of ODEs and describe in detail the different types of force of infection terms used in our model. Then, we present our parameter estimation, the simulation results, and predict how changes in certain parameters impact the UTCVM population. Our model is data-driven using both the COVID-19 cases in Knox county and within the UTCVM community. This novel predictive model was critical in assessing risk and guiding intervention strategies for UTCVM's smooth and safe operation.

## 2 Model formulation

To explain the model, we first provide more information about the situation at the UTCVM clinic. In March 2020, both clinical and pre-clinical students at UTCVM were sent home after spring break to complete the semester online. All personnel remaining in the UTCVM building were required to wear masks, and all employees were subjected to strict travel restrictions. Clients were not permitted to enter the building and a “drop off/pick up” system was devised to allow clients bring their animals to the hospital. Cleaning and disinfection protocols were enhanced throughout the building, and all employees who could work from home were asked to do so. An internal contact tracing team was created to allow rapid identification of sick or exposed individuals in the college. Prevention of intra-hospital spread was the primary goal of the enacted safety protocols. After approximately six weeks of only emergent/urgent care and no hospital-associated cases of COVID-19 in on-duty personnel, UTCVM services began to gradually increase patient receiving while maintaining the prohibition of client entry into the facility. Students also were gradually returned to the hospital to continue their education with in-person, hybrid and online versions of clinical rotations; after approximately four months (in mid-September), clinical rotations returned to fully in-person instruction of students.

A daily self-screening tool was developed for all University of Tennessee faculty, staff, and students to use in order to complete a self-screening questionnaire. A positive response (“yes”) to any question would trigger an alert to the supervisor and the UT Emergency Operations Committee, and that individual would be provided self-isolation instructions. Individuals placed in isolation were contacted by college personnel to determine the reason for isolation and additional steps were taken as needed (Peak et al., 2020).

Incidence data on college COVID-19 cases, close contacts of cases, and identification of intra-college spread of disease were all continuously tracked. Data was reviewed weekly by the college's COVID-19 task force, and safety protocols were adjusted as needed to ensure employee and student safety. The first case of COVID-19 due to intra-hospital transmission occurred on August 4, 2020 and the infected individual fully recovered. As the state of Tennessee relaxed some of the initial restrictions imposed at the onset phase of the pandemic, local veterinarians requested that the UTCVM hospital increase the number of services offered for patient referral. Opening more client services would lead to increased human density within the UTCVM building or increased contact time between clients and UTCVM personnel. See Table 1 which contains key dates about the UTCVM response.

**Table 1:** Timeline of the UTCVM response to COVID-19.

Date	Event
March 19, 2020	UTCVM begins handling urgent cases only
April 30, 2020	UTCVM begins to gradually increase caseloads
June 2020	UTCVM begins to gradually transition to full time instruction of students
September 2020	UTCVM has transitioned to full time instruction of students

Our approach uses a system of ordinary differential equations, with the population split into two social groups: (1) Staff and clinicians who work at the UTCVM and (2) Knox county community. In the model, the total population is divided into 10 compartments, where the subscript *v* represents staff that work in the clinic and the subscript *p* represents the Knox county community. We use a Lagrangian approach that has been modified to use the time spent in the clinic by UTCVM personnel and separately from the time spent at the clinic by the Knox county community. Our adaptation is required because the clinic is not a residence, but rather a location with a fixed number of staff/clinician and clients visiting each day.

Following a modified SEIR approach with some Lagrangian features, the compartments represent: *S* susceptible individuals, *E* exposed individuals, *A* asymptomatic individuals, *I* pre-symptomatic and symptomatic individuals, *Q* individuals with confirmed infections, and *R* recovered individuals. Note that we make a distinction between pre-symptomatic individuals and asymptomatic individuals. Here, asymptomatic individuals are individuals who remain asymptomatic throughout the disease process, while pre-symptomatic individuals are infected individuals who are not presently symptomatic but will later become symptomatic. We make this distinction because pre-symptomatic individuals transmit the infection at a rate similar to symptomatic individuals, and asymptomatic individuals transmit the infection at a much lower rate than pre-symptomatic and symptomatic individuals (Centers for Disease Control and Prevention, 2021; Edholm et al., 2022).

As a result, we assume that an infectious symptomatic or pre-symptomatic individual is more likely to transmit COVID-19 than an asymptomatic individual. To account for this, we multiplied the contact rate within the force of infection by *b* > 1. Furthermore, because all UTCVM staff and clinicians were pre-screened for COVID-19 risk factors before entering the clinic, some asymptomatic *A*_*v*_ may test positive and then must self-isolate. As a result, our model moves asymptomatic UTCVM personnel to the *Q* compartment at rate *γ*.

Furthermore, we assume that infected individuals in the Knox county community may choose not to be tested for COVID-19 despite exhibiting symptoms, which is why our model allows individuals to move directly from the *I*_*p*_ compartment to the recovered *R* compartment. In contrast, we assume that members of the UTCVM community are vigilant about COVID-19 and are tested if they develop symptoms, and so our model does not allow an individual in the *I*_*v*_ compartment to move directly into the *R* compartment. Due to the lack of wide-spread testing of asymptomatic individuals in Knox county, we do not include a route for asymptomatic individuals *A*_*p*_ to transition directly into the *Q* class.

In our model, we have natural birth rates Γ_1_, Γ_2_ for *S*_*v*_, *S*_*p*_ respectively and a natural death rate *d*. We use these rates to calculate the basic reproductive number, *R*_0_, but we do not use them for the numerical simulations due to the short time frame used for the simulations.

**Fig. 1:**
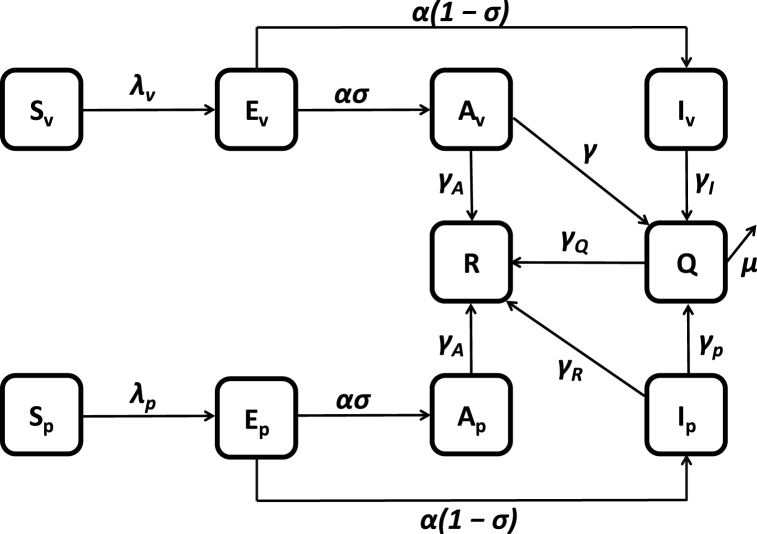
Diagram of the flow through the UTCVM classes in our model with parameters as in Table 2 and with the force of infection terms acting on *S*_*v*_ denoted by *λ*_*v*_, and the force of infection terms acting on *S*_*p*_ denoted *λ*_*p*_.

Our model with the given compartments is below:

$$\begin{array}{cc} \frac{dS_{v}}{dt} & =\Gamma_{1}-v_{1}\left[\frac{\beta_{vv}v_{1}\left(A_{v}+bI_{v}\right)}{v_{1}N_{v}}+\frac{\beta_{vp}\left(1+c\right)p_{1}\left(A_{p}+bI_{p}\right)}{v_{1}N_{v}+p_{1}N_{p}}\right]S_{v}-\left(1-v_{1}\right)\left[\frac{\beta_{vp}\left(1-p_{1}\right)\left(A_{p}+bI_{p}\right)}{\left(1-p_{1}\right)N_{p}+\left(1-v_{1}\right)N_{v}}\right]S_{v}-dS_{v}\\ \frac{dS_{p}}{dt} & =\Gamma_{2}-\left(1-p_{1}\right)\left[\frac{\beta_{pp}\left(1-p_{1}\right)\left(A_{p}+bI_{p}\right)}{\left(1-p_{1}\right)N_{p}+\left(1-v_{1}\right)N_{v}}\right]S_{p}-p_{1}\frac{\beta_{vp}v_{1}\left(A_{v}+bI_{v}\right)}{v_{1}N_{v}}S_{p}-dS_{p}\\ \frac{dE_{v}}{dt} & =v_{1}\left[\frac{\beta_{vv}v_{1}\left(A_{v}+bI_{v}\right)}{v_{1}N_{v}}+\frac{\beta_{vp}\left(1+c\right)p_{1}\left(A_{p}+bI_{p}\right)}{v_{1}N_{v}+p_{1}N_{p}}\right]S_{v}+\left(1-v_{1}\right)\left[\frac{\beta_{vp}\left(1-p_{1}\right)\left(A_{p}+bI_{p}\right)}{\left(1-p_{1}\right)N_{p}+\left(1-v_{1}\right)N_{v}}\right]S_{v}-dE_{v}-\alpha E_{v}\\ \frac{dE_{p}}{dt} & =\left(1-p_{1}\right)\left[\frac{\beta_{pp}\left(1-p_{1}\right)\left(A_{p}+bI_{p}\right)}{\left(1-p_{1}\right)N_{p}+\left(1-v_{1}\right)N_{v}}\right]S_{p}+p_{1}\frac{\beta_{vp}v_{1}\left(A_{v}+bI_{v}\right)}{v_{1}N_{v}}S_{p}-dE_{p}-\alpha E_{p}\\ \frac{dA_{v}}{dt} & =\alpha \sigma E_{v}-\gamma_{A}A_{v}-\gamma A_{v}-dA_{v}\\ \frac{dA_{p}}{dt} & =\alpha \sigma E_{p}-\gamma_{A}A_{p}-dA_{p}\\ \frac{dI_{v}}{dt} & =\alpha \left(1-\sigma \right)E_{v}-\gamma_{I}I_{v}-dI_{v}\\ \frac{dI_{p}}{dt} & =\alpha \left(1-\sigma \right)E_{p}-\left(\gamma_{p}+\gamma_{R}\right)I_{p}-dI_{p}\\ \frac{dQ}{dt} & =\gamma A_{v}+\gamma_{I}I_{v}+\gamma_{p}I_{p}-\left(\gamma_{Q}+\mu \right)Q-dQ\\ \frac{dR}{dt} & =\gamma_{A}\left(A_{v}+A_{p}\right)+\gamma_{R}I_{p}+\gamma_{Q}Q-dR \end{array}$$

where *N*_*v*_ = *S*_*v*_ + *E*_*v*_ + *A*_*v*_ + *I*_*v*_ and *N*_*p*_ = *S*_*p*_ + *E*_*p*_ + *A*_*p*_ + *I*_*p*_ + *R*. The system is illustrated by Fig. 1 with the descriptions of the parameters given in Table 2.

**Table 2:** Our parameters, their interpretations, values, and sources. As described in the text, the parameter *β*_*vp*_ is time dependent, with two values and the switch occurring in October.

	Parameter Interpretation	Value	Source
*β*_*vv*_	Transmission coefficient among clinicians in clinic	0.6398	Fitted
*β*_*pp*_	Transmission coefficient among county community	0.1728	Fitted
*β*_*vp*_	Transmission coefficient from *A*_*p*_, *I*_*p*_ to *S*_*v*_ (scaled by (1 + *c*)) in clinic and from *A*_*p*_, *I*_*p*_ to *S*_*v*_ outside the clinic	0.03, 0.05	Fitted
*b*	Parameter used to increase the transmission rate of *I*_*v*_, *I*_*p*_, as compared to that of *A*_*v*_, *A*_*p*_	3.0142	Fitted
*c*	Parameter used to increase transmission rate from *A*_*p*_, *I*_*p*_ to *S*_*v*_ while in clinic	0.9814	Fitted
*γ*_*R*_	Recovery rate of *I*_*p*_	0.1878	Fitted
*γ*	Transition from *A*_*v*_ to *Q*	0.0606	Fitted
*γ*_*I*_	Transition rate from *I*_*v*_ to *Q*	0.155	Fitted
*α*	Transition rate from *E*_*v*_, *E*_*p*_ to corresponding *A*, *I*	0.33	Sanche et al.
*σ*	Fraction of *E*_*v*_, *E*_*p*_ becoming *A*_*v*_, *A*_*p*_	0.4	Oran and Topol (2021)
*γ*_*p*_	Transition rate from *I*_*p*_ to *Q*	1/7	Renardy et al. (2020)
*γ*_*Q*_	Transition rate from *Q* to *R*	1/10	Renardy et al. (2020)
*γ*_*A*_	Transition rate from *A*_*v*_, *A*_*p*_ to *R*	1/4	Agusto et al. (2022)
*μ*	Death rate out of *Q* due to disease	0.0065	Centers for Disease Control and Prevention (2021)
*a*_*p*_	Knox county population	470000	United States Census Bureau (2021)
*d*_*p*_	Number of clients in vet clinic per day	60	UTCVM
*a*_*v*_	Total number of clinicians at vet clinic	300	UTCVM
*d*_*v*_	Number of clinicians in clinic per day	50	UTCVM

In our adapted Lagrangian model, the terms in the forces of infection are grouped by

$$\begin{array}{cc} \lambda_{v} & =v_{1}\left[\frac{\beta_{vv}v_{1}\left(A_{v}+bI_{v}\right)}{v_{1}N_{v}}+\frac{\beta_{vp}\left(1+c\right)p_{1}\left(A_{p}+bI_{p}\right)}{v_{1}N_{v}+p_{1}N_{p}}\right] \end{array}+\left(1-v_{1}\right)\left[\frac{\beta_{vp}\left(1-p_{1}\right)\left(A_{p}+bI_{p}\right)}{\left(1-p_{1}\right)N_{p}+\left(1-v_{1}\right)N_{v}}\right]$$

and

$$\lambda_{p}=\left(1-p_{1}\right)\left[\frac{\beta_{pp}\left(1-p_{1}\right)\left(A_{p}+bI_{p}\right)}{\left(1-p_{1}\right)N_{p}+\left(1-v_{1}\right)N_{v}}\right]+p_{1}\frac{\beta_{vp}v_{1}\left(A_{v}+bI_{v}\right)}{v_{1}N_{v}}.$$

We now describe the meaning of *λ*_*v*_ and *λ*_*p*_ in detail. In our model, the terms *v*_1_ and *p*_1_ represent the proportion of time that the UTCVM personnel and the Knox county community spend at the clinic, respectively. Explicitly, these terms are defined as

$$v_{1}=\frac{1}{2}\frac{d_{v}}{a_{v}}\;p_{1}=\frac{1}{24}\frac{d_{p}}{a_{p}}$$

The proportion *v*_1_ captures the assumption that the UTCVM clinicians and staff spend 1/2 at the clinic on days they work. The fraction *d*_*v*_/*a*_*v*_ captures the proportion of clinicians and staff who are present on any given day, where *d*_*v*_ is the number of clinicians and staff present in the clinic and *a*_*v*_ is the total number of clinicians and staff who work at UTCVM. This fraction is necessary as not all staff members work at UTCVM each day. Note *a*_*v*_ is approximately equal to *N*_*v*_ = *S*_*v*_ + *E*_*v*_ + *A*_*v*_ + *I*_*v*_ due to the very low number of deaths due to COVID-19 in the UTCVM population.

Analogously, the proportion *p*_1_ represents the proportion of time that the Knox county community spends at the clinic. We assume that the public spends 1 h (1/24) when they are at the clinic. Since not all members of the public will need to visit the clinic on a given day, we multiply by *d*_*p*_/*a*_*p*_, where *d*_*p*_ is the number of clients served at the clinic per day, and *a*_*p*_ is the Knox county population at the start of this model. Note that *N*_*p*_ = *S*_*p*_ + *E*_*p*_ + *A*_*p*_ + *I*_*p*_ + *R* could change over time due to deaths in the community.

We now describe the meaning of each of the terms represented in *λ*_*v*_. The first term represents intra-hospital transmission of COVID-19 among UTCVM personnel, from *A*_*v*_, *I*_*v*_ to *S*_*v*_:

$$v_{1}\left[\frac{\beta_{vv}v_{1}\left(A_{v}+bI_{v}\right)}{v_{1}N_{v}}\right]S_{v}.$$

The second term involved in *λ*_*v*_ gives the transmission to *S*_*v*_ from infected public clients, *A*_*p*_, *I*_*p*_ who are visiting the clinic:

$$v_{1}\left[\frac{\beta_{vp}\left(1+c\right)p_{1}\left(A_{p}+bI_{p}\right)}{v_{1}N_{v}+p_{1}N_{p}}\right]S_{v}.$$

The parameter *c* represents the increased transmission due to the effect of intentional appointments at UTCVM rather than random encounters. The third term involved in *λ*_*v*_ gives the transmission of COVID-19 to *S*_*v*_ while in the community (outside of the clinic):

$$\left(1-v_{1}\right)\left[\frac{\beta_{vp}\left(1-p_{1}\right)\left(A_{p}+bI_{p}\right)}{\left(1-p_{1}\right)N_{p}+\left(1-v_{1}\right)N_{v}}\right]S_{v}.$$

This relies on the assumption that the transmission of COVID-19 between UTCVM personnel is negligible outside of the clinic.

We now discuss the meaning of the two terms in *λ*_*p*_. The first term

$$\left(1-p_{1}\right)\left[\frac{\beta_{pp}\left(1-p_{1}\right)\left(A_{p}+bI_{p}\right)}{\left(1-p_{1}\right)N_{p}+\left(1-v_{1}\right)N_{v}}\right]S_{p}$$

Represents the transmission of COVID-19 to *S*_*p*_ from *A*_*p*_ and *I*_*p*_ at locations other than the clinic. The second term

$$p_{1}\frac{\beta_{vp}v_{1}\left(A_{v}+bI_{v}\right)}{v_{1}N_{v}}S_{p}$$

Represents transmission to *S*_*p*_ from infected members of UTCVM. Since members of the public are not allowed to enter UTCVM and hence have no contact with other members of the public when visiting UTCVM, we intentionally do not have a transmission route between members of the public while visiting the UTCVM location. Furthermore, there is not a term for the infected members of the UTCVM community to transmit to the Knox county community outside of the clinic because we assume that transmission route to be negligible due to the vigilance of the UTCVM community.

## 3 Data and parameter estimation

We fit our model to both Knox county infection data and UTCVM infection data. We fit our model to Knox county data that began on July 28, 2020 and ended on March 18, 2021. The UTCVM data began on July 28, 2020 and ended on February 10, 2021. We used daily time steps for both data sets when fitting our model. Our Knox county data are taken from the Knox County COVID-19 Case Count Reports, available online during the pandemic (Knox County, 2021). The UTCVM data were obtained internally and are given in Appendix A. We used cumulative confirmed infection data throughout the parameter estimation process.

Some parameters were chosen from the literature, while eight others (mostly transmission related) were estimated from the data; this is summarized in Table 2. We estimated these parameters by creating a least-squares optimization problem, where the goal was to minimize the difference of the daily cumulative confirmed cases within Knox county and UTCVM and our model's simulation. The objective function to be minimized is given by

$$\frac{\Sigma_{i}{\left(C_{i}-Data_{i}\right)}^{2}}{\Sigma_{i}{\left(Data_{i}\right)}^{2}}+\frac{\Sigma_{i}{\left(VC_{i}-VData_{i}\right)}^{2}}{\Sigma_{i}{\left(VData_{i}\right)}^{2}},$$

where *C*_*i*_ and *Data*_*i*_ represent the cumulative number of confirmed cases within Knox county from the simulation and from the Knox county data. Similarly, *VC*_*i*_ and *VData*_*i*_ represent the cumulative number of confirmed cases in UTCVM, from simulation and in the data.

To solve this least-squares optimization problem, we used MATLAB version R2022a and the functions MultiStart and fmincon. These functions, included in the MATLAB optimization and global optimization toolboxes, perform a search over the parameter space to minimize the objective function.

We first fit the data by estimating the parameters to be constant over our time frame, but we were not able to satisfactorily capture the dynamics within the UTCVM compartments, due to the rise in the cases in November. This resulted in the simulated cumulative confirmed cases for UTCVM underestimating the actual values.

**Fig. 2:**
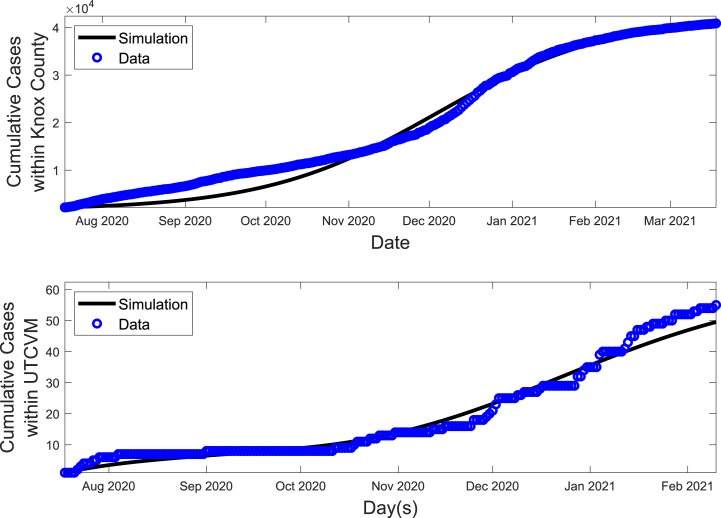
Results from fitting the model to the COVID-19 data given in A.3 and in (Knox County, 2021).

Because of changes in behavior and/or government mandates regarding social distancing, some rates in other COVID-19 models have been chosen to be time-dependent (Edholm et al., 2022; Eikenberry et al., 2020). Therefore, we investigated the impact of holding those parameters constant over time except for the transmission rates *β*_*vp*_. By examining the resulting simulations and corresponding errors, we increased *β*_*vp*_ from 0.03 to 0.05, with this change starting in October. Then, the transition between the two values of *β*_*vp*_ was smoothed using interpolation. This resulted in a far better fit for the cumulative UTCVM infections with relative error 0.1807. Our decision to increase *β*_*vp*_ in October is supported by some relaxation of the local COVID-19 restrictions in Knox county at that time and by the effects of more interactions in the UTCVM clinic and the corresponding student activities. These parameters were used in the simulations to illustrate dynamics in our compartments and calculate the relative errors in our parameter fitting. We compare our model simulation to the daily cumulative UTCVM and Knox county data in Fig. 2. As we are aware of the need to consider weekly data in addition to cumulative data (King et al., 2015), we have also produced a plot that compares our model's simulation to weekly cumulative data in Fig. 4, Fig. 5. This shows that our model fits reasonably well to the weekly cumulative data.

## 4 Results

**Fig. 3:**
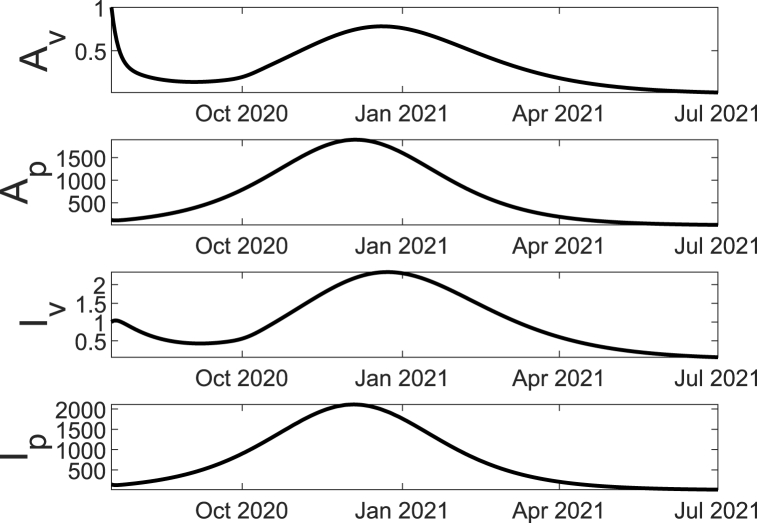
Simulations for the infected compartments using our estimated parameters, starting on July 18, 2020.

Using our parameters shown in Table 2, the dynamics of the infected compartments are shown in Fig. 3. Note all those compartments have their peaks in December 2020, but the peaks in *A*_*p*_, *I*_*p*_ happened shortly before those in *A*_*v*_, *I*_*v*_. These simulations started on July 18, 2020.

**Fig. 4:**
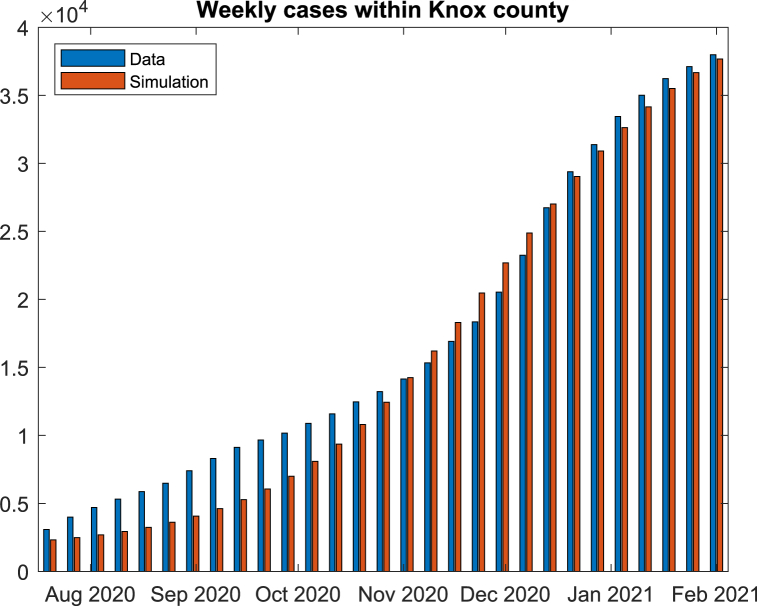
Simulation output of the confirmed weekly cases within Knox county is plotted alongside the confirmed weekly case data given in (Knox County, 2021). This shows that the model fits reasonably well to the weekly infection data.

**Fig. 5:**
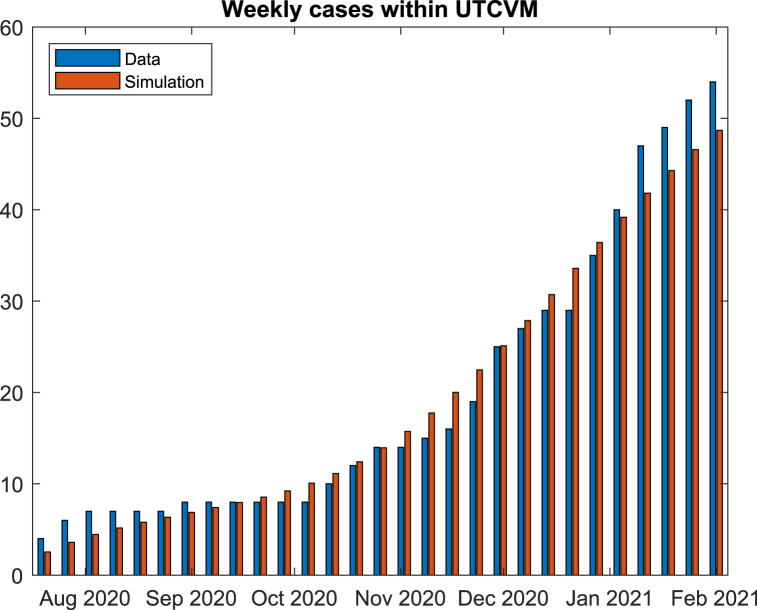
Simulation output of the confirmed weekly cases within the UTCVM susceptibles *S*_*v*_ is plotted alongside the confirmed weekly case data given in Appendix A. This shows that the model fits reasonably well to the weekly infection data.

Then, we simulated various scenarios that could be experienced by changes in possible re-opening actions of the clinic and behavior changes within Knox county. Each of these scenarios were started on July 18, 2020 and with the parameter of interest changing on April 1, 2021. For the other parameters, we used their estimated values as given in Table 2.

**Fig. 6:**
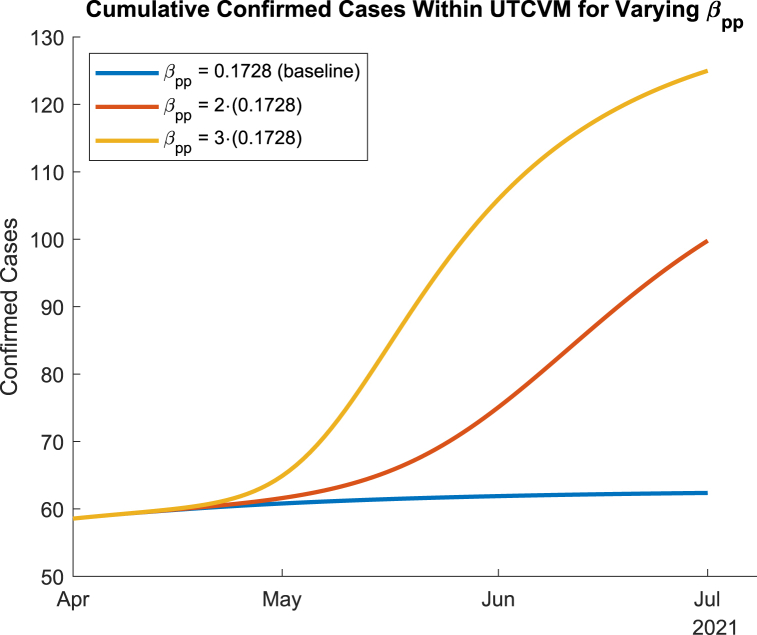
This plot simulates how the quantity of confirmed infections coming from *S*_*v*_ is impacted by changes in the parameter *β*_*pp*_. This shows that the UTCVM community is strongly impacted by behavior changes in Knox county. In this simulation, the parameter *β*_*pp*_ was changed on April 1, 2021.

To understand how *S*_*v*_ is impacted by changes within the Knox county community, we considered the effect of increasing the parameter *β*_*pp*_. These results are shown in Fig. 6. We can see that increases in transmission among members of the public increases the number of confirmed cases within the veterinary clinic community *S*_*v*_.

**Fig. 7:**
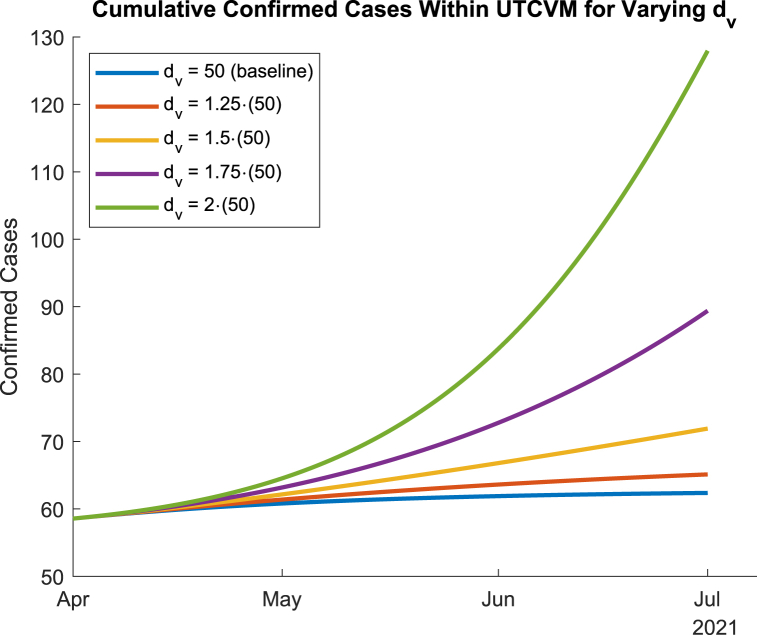
This plot simulates how the quantity of confirmed infections from *S*_*v*_ is impacted by changes to the parameter *d*_*v*_. This corresponds with increasing staffing within the clinic, while keeping other parameters the same. In this simulation, the parameter *β*_*pp*_ was changed on April 1, 2021.

We now consider how the parameter *d*_*v*_ impacts COVID-19 transmission, which corresponds to increasing the amount of staff working in the clinic. An example of how increasing the parameter *d*_*v*_ is impacts disease transmission to *S*_*v*_ is shown in Fig. 7. In particular, we note that doubling *d*_*v*_ results in over double the number of infections.

**Fig. 8:**
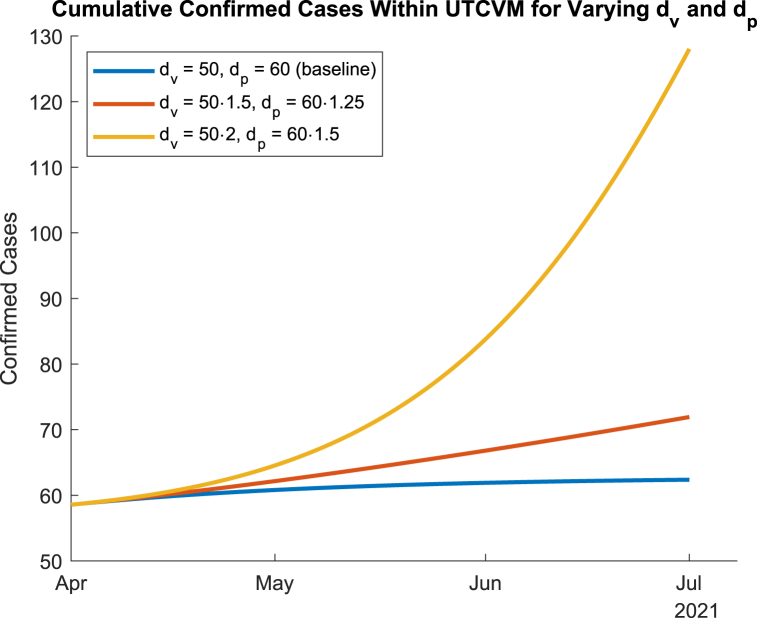
This plot simulates how the quantity of confirmed infections from *S*_*v*_ is impacted by changes to the parameter *d*_*v*_ and *d*_*p*_ changing simultaneously. This simulation corresponds with increasing both staffing within the UTCVM and the number of animals treated at the clinic (and their owners). Both parameters were changed on April 1, 2021 in this simulation.

Similarly, we consider how increasing *d*_*p*_ impacts the infection rate to *S*_*v*_. We found that increasing *d*_*p*_ has no meaningful impact on the number of infections in *S*_*v*_. Increasing both *d*_*v*_ and *d*_*p*_ simultaneously does lead to significantly increased infection to *S*_*v*_, however, the increase is primarily due to the change in *d*_*v*_. But when the number of clients *d*_*p*_ increases, the number of clinic personnel *d*_*v*_ needs to increase to give service and treatments to the additional animals of the clients. This behavior is shown in Fig. 8.

We numerically derived the basic reproductive number *R*_0_ using the standard Next Generation Matrix method (Van den Driessche & Watmough, 2002; Diekmann et al., 2010). The details including the disease-free equilibrium vector and the matrix computations are included in Appendix B. For our parameter set, we found that the basic reproductive number to be *R*_0_ = 1.18. This number gives the expected number of infections generated by a single infectious individual in a fully susceptible population.

## 5 Discussion

The operation of the UTCVM veterinary teaching hospital was deemed essential and steps were taken to ensure personnel safety, adequate training of students, and continued patient care. Numerous safety measures were instituted when COVID-19 was declared a pandemic and local cases were identified. As local case counts varied, the implemented safety measures were altered to allow continued hospital operation.

Mathematically, our model was able to represent the change in certain transmission rates in November, to give a good fit to the data and to show appropriate dynamics in our compartments. The peaks of the infectious classes occurred in late November, which can be seen both in our simulation plots (Fig. 3) and in the rise of weekly cases at that time (Fig. 4).

Our results about varying *d*_*v*_, *d*_*p*_, and *β*_*pp*_ show that risk to UTCVM personnel is strongly impacted by both policies controlled directly by UTCVM leadership and changes within Knox county. Note that *β*_*pp*_ is affected by government mandates and closings in the county. This means that UTCVM leadership must be vigilant to protect its clinicians and staff, and react quickly to various scenarios that may change the behavior of the public.

For our model and parameter set (with *β*_*vp*_ = 0.03), the basic reproductive number, *R*_0_ = 1.14, means that the disease-free equilibrium is unstable and thus the number of infected individuals would not tend to 0 as time goes on, without further interventions or changes in behavior (Van den Driessche & Watmough, 2002).

## 6 Conclusion

This model was developed to determine the impact of various safety measures put in place to prevent intra-hospital spread of COVID-19 before widely available vaccination. When considering veterinary hospital operations during any future emerging infectious disease outbreaks, public health preventive strategies must often be the first line of defense as vaccinations may not be available for novel pathogens.

Better understanding of the spread of COVID-19 in a clinical environment is important to inform policy decisions. Our work quantifies the risk of various re-opening scenarios that UTCVM could consider during the global pandemic. Our model has novel features of two populations (the public and the veterinary staff, students, and clinicians) in which only a small proportion of the public population interacts with the vet staff/clinicians for a short time each day. This situation is quite different from medical hospitals, in which the patients may stay for much longer periods. We used an adapted Lagrangian-type model to represent our situation, which was different from models that concern human medical hospitals, such as those in (Evans et al., 2021; Gozalpour et al., 2021).

Our results confirmed that more clients and staff in the clinic will increase the confirmed cases in the UTCVM population. The cumulative cases in the UTCVM population could double if some contact rates (representing the public interactions) in *β*_*pp*_ are increased. Increasing the number of clients and clinicians in the clinic each day gives also substantial increases in the number of confirmed cases in the UTCVM population.

The *R*_0_ value predicts the continuation of the outbreak with the bio-safety practices in mid-summer 2020. Thus, this shows a need for further interventions, such as vaccination and increased testing.

During the initial analysis of this paper, the Omicron variant of COVID-19 had begun to surface. Future work could modify this model to factor in the changing epidemiological properties of the newer variants and how the infection rate is impacted by progressively relaxed restrictions within Knox county and the increased availability of vaccination. Furthermore, considering demographic and socioeconomic features that affect COVID-19 cases may be valuable (Igoe et al., 2022). A limitation of our model is that the UTCVM population is small when compared to the county population, and future work could utilize a discrete model (which may more accurate with small populations) and possibly include stochastic features.

## Declaration of competing interest

The authors certify that there is no conflict of interest.

## Figures and Tables

**Table A.3:** UTCVM COVID-19 data, new and cumulative cases.

Date	New	Cumulative
7/18/2020	1	1
7/22/2020	1	2
7/23/2020	1	3
7/24/2020	1	4
7/27/2020	1	5
7/29/2020	1	6
8/4/2020	1	7
9/1/2020	1	8
10/12/2020	1	9
10/18/2020	1	10
10/19/2020	1	11
10/23/2020	1	12
10/26/2020	1	13
10/31/2020	1	14
11/12/2020	1	15
11/16/2020	1	16
11/25/2020	1	18
11/29/2020	1	19
11/30/2020	1	20
12/1/2020	1	21
12/2/2020	2	23
12/3/2020	2	25
12/9/2020	1	26
12/11/2020	1	27
12/16/2020	1	28
12/17/2020	1	29
12/28/2020	3	32
12/30/2020	2	34
12/31/2020	1	35
1/4/2021	3	39
1/5/2021	1	40
1/12/2021	1	41
1/13/2021	2	43
1/14/2021	2	45
1/16/2021	2	47
1/19/2021	1	48
1/21/2021	1	49
1/25/2021	1	50
1/28/2021	2	52
2/3/2021	1	53
2/5/2021	1	54
2/10/2021	1	55

## Appendix B Disease-Free equilibrium and the calculation of *R*_0_

The disease-free equilibrium (DFE) for our model is given by:

$$X^{*}=\left(S_{v}^{*},S_{p}^{*},0,0,0,0,0,0,0,0\right)=\left(\frac{\Gamma_{1}}{d},\frac{\Gamma_{2}}{d},0,0,0,0,0,0,0,0\right)$$

To investigate the stability of the disease-free equilibrium, we use the Next Generation Matrix method developed in (Van den Driessche & Watmough, 2002) to calculate the basic reproductive number $R_{0}$. We consider *E*_*v*_, *E*_*p*_, *A*_*v*_, *A*_*p*_, *I*_*v*_, *I*_*p*_. as infected compartments and *S*_*v*_, *S*_*p*_, *Q*, *R* as noninfected compartments. Note that *Q* is an isolated compartment. First, we find the rates of new infections for all infected compartments, and call this F.

$$\left[\begin{array}{c} v_{1}\left[\frac{\beta_{vv}v_{1}\left(A_{v}+bI_{v}\right)}{v_{1}N_{v}}+\frac{\beta_{vp}\left(1+c\right)p_{1}\left(A_{p}+bI_{p}\right)}{v_{1}N_{v}+p_{1}N_{p}}\right]S_{v}+\left(1-v_{1}\right)\left[\frac{\beta_{vp}\left(1-p_{1}\right)\left(A_{p}+bI_{p}\right)}{\left(1-p_{1}\right)N_{p}+\left(1-v_{1}\right)N_{v}}\right]S_{v}\\ \left(1-p_{1}\right)\left[\frac{\beta_{pp}\left(1-p_{1}\right)\left(A_{p}+bI_{p}\right)}{\left(1-p_{1}\right)N_{p}+\left(1-v_{1}\right)N_{v}}\right]S_{p}+p_{1}\frac{\beta_{vp}v_{1}\left(A_{v}+bI_{v}\right)}{v_{1}N_{v}}S_{p}\\ 0\\ 0\\ 0\\ 0 \end{array}\right]$$

Then, we find the rates of transition for the infected compartments and call this V.

$$\left[\begin{array}{c} \alpha E_{v}+dE_{v}\\ \alpha E_{p}+dE_{p}\\ -\alpha \sigma E_{v}+\gamma_{A}A_{v}+\gamma A_{v}+dA_{v}\\ -\alpha \sigma E_{p}+\gamma_{A}A_{p}+dA_{p}\\ -\alpha \left(1-\sigma \right)E_{v}+\gamma_{I}I_{v}+dI_{v}\\ -\alpha \left(1-\sigma \right)E_{p}+\left(\gamma_{I}+\gamma_{R}\right)I_{p}+dI_{p} \end{array}\right]$$

We calculate the Jacobian matrix from new infections, **F**.

$$F\left(X^{*}\right)=\left[\begin{array}{cccccc} 0 & 0 & f_{1} & f_{2} & f_{3} & f_{4}\\ 0 & 0 & f_{5} & f_{6} & f_{7} & f_{8}\\ 0 & 0 & 0 & 0 & 0 & 0\\ 0 & 0 & 0 & 0 & 0 & 0\\ 0 & 0 & 0 & 0 & 0 & 0\\ 0 & 0 & 0 & 0 & 0 & 0 \end{array}\right]$$

with

$$\begin{array}{l} f_{1}=v_{1}\beta_{vv}\\ f_{2}=\frac{v_{1}S_{v}^{*}\beta_{vp}\left(1+c\right)p_{1}}{v_{1}S_{v}^{*}+p_{1}S_{p}^{*}}+\frac{\left(1-v_{1}\right)S_{v}^{*}\beta_{vp}\left(1-p_{1}\right)}{\left(1-p_{1}\right)S_{p}^{*}+\left(1-v_{1}\right)S_{v}^{*}}\\ f_{3}=v_{1}b\beta_{vv}\\ f_{4}=\frac{v_{1}S_{v}^{*}\beta_{vp}\left(1+c\right)p_{1}}{v_{1}S_{v}^{*}+p_{1}S_{p}^{*}}+\frac{\left(1-v_{1}\right)S_{v}^{*}\beta_{vp}\left(1-p_{1}\right)}{\left(1-p_{1}\right)S_{p}^{*}+\left(1-v_{1}\right)S_{v}^{*}}\\ f_{5}=\frac{\beta_{vp}p_{1}S_{p}^{*}}{S_{v}^{*}}\\ f_{6}=\frac{\left(1-p_{1}\right)\beta_{pp}\left(1-p_{1}\right)S_{p}^{*}}{\left(1-p_{1}\right)S_{p}^{*}+\left(1-v_{1}\right)S_{v}^{*}}\\ f_{7}=\frac{p_{1}\beta_{vp}bS_{p}^{*}}{S_{v}^{*}}\\ f_{8}=\frac{\left(1-p_{1}\right)\beta_{pp}\left(1-p_{1}\right)bS_{p}^{*}}{\left(1-p_{1}\right)S_{p}^{*}+\left(1-v_{1}\right)S_{v}^{*}}. \end{array}$$

We calculate the Jacobian matrix of V, called **V**.

$$V\left(X^{*}\right)=\left[\begin{array}{cccccc} v_{1} & 0 & 0 & 0 & 0 & 0\\ 0 & v_{1} & 0 & 0 & 0 & 0\\ v_{2} & 0 & v_{3} & 0 & 0 & 0\\ 0 & v_{2} & 0 & v_{4} & 0 & 0\\ v_{5} & 0 & 0 & 0 & v_{6} & 0\\ 0 & v_{5} & 0 & 0 & 0 & v_{7} \end{array}\right]$$

with *v*_1_ = *α* + *d*, *v*_2_ = −*ασ*, *v*_3_ = *γ*_*A*_ + *γ* + *d*, *v*_4_ = *γ*_*A*_ + *d*, *v*_5_ = −*α*(1 − *σ*), *v*_6_ = *γ*_*I*_ + *d*, *v*_7_ = *γ*_*I*_ + *γ*_*R*_ + *d*. We need to calculate the inverse of **V** to find the Next Generation Matrix.

$$V^{-1}=\left[\begin{array}{cccccc} \frac{1}{v_{1}} & 0 & 0 & 0 & 0 & 0\\ 0 & \frac{1}{v_{1}} & 0 & 0 & 0 & 0\\ -\frac{v_{2}}{v_{1}v_{3}} & 0 & \frac{1}{v_{3}} & 0 & 0 & 0\\ 0 & -\frac{v_{2}}{v_{1}v_{4}} & 0 & \frac{1}{v_{4}} & 0 & 0\\ -\frac{v_{5}}{v_{1}v_{6}} & 0 & 0 & 0 & \frac{1}{v_{6}} & 0\\ 0 & -\frac{v_{5}}{v_{1}v_{7}} & 0 & 0 & 0 & \frac{1}{v_{7}} \end{array}\right]$$

The product **FV**^**−1**^ at the DFE gives us the Next Generation Matrix and the spectral radius of this matrix gives the basic reproductive number $R_{0}$ which is a threshold value for the DFE to be locally stable:

$$FV^{-1}\left(X^{*}\right)=\left[\begin{array}{cccccc} n_{1} & n_{2} & n_{3} & n_{4} & n_{5} & n_{6}\\ n_{7} & n_{8} & n_{9} & n_{10} & n_{11} & n_{12}\\ 0 & 0 & 0 & 0 & 0 & 0\\ 0 & 0 & 0 & 0 & 0 & 0\\ 0 & 0 & 0 & 0 & 0 & 0\\ 0 & 0 & 0 & 0 & 0 & 0 \end{array}\right]$$

with

$$\begin{array}{l} n_{1}=-\frac{v_{6}v_{2}f_{1}+f_{3}v_{5}v_{3}}{v_{1}v_{3}v_{6}}\\ n_{2}=-\frac{v_{7}v_{2}f_{2}+v_{4}v_{5}f_{4}}{v_{1}v_{4}v_{7}}\\ n_{3}=\frac{f_{1}}{v_{3}}\\ n_{4}=\frac{f_{2}}{v_{4}}\\ n_{5}=\frac{f_{3}}{v_{6}}\\ n_{6}=\frac{f_{4}}{v_{7}}\\ n_{7}=-\frac{v_{6}v_{2}f_{5}+f_{7}v_{5}v_{3}}{v_{1}v_{3}v_{6}}\\ n_{8}=-\frac{v_{7}v_{2}f_{6}+v_{4}v_{5}f_{8}}{v_{1}v_{4}v_{7}}\\ n_{9}=\frac{f_{5}}{v_{3}}\\ n_{10}=\frac{f_{6}}{v_{4}}\\ n_{11}=\frac{f_{7}}{v_{6}}\\ n_{12}=\frac{f_{8}}{v_{7}}. \end{array}$$

Thus, we obtain

$$R_{0}=\frac{n_{1}+n_{8}+\sqrt{n_{1}^{2}+4n_{2}n_{7}-2n_{1}n_{8}+n_{8}^{2}}}{2}$$

If this value is less than one, then the DFE is locally stable, otherwise the DFE is unstable.

Note that in calculating *R*_0_ numerically, we used *β*_*vp*_ = 0.03, *d* = 2.3562 ⋅ 10^−5^, Γ_1_ = 14.6488 ⋅ (300/470000), Γ_2_ = 14.6488 (Tennessee Department of Health, 2022), *β*_*vp*_ = 0.03, in addition to the parameters in Table 2.
