# Supplementary material for: The effect of changing COVID-19 restrictions on the transmission rate in a veterinary clinic
Source: Infect Dis Model. 2023 Feb 10;8(1):294–308. doi: 10.1016/j.idm.2023.01.005 (PMC9916190; doi:10.1016/j.idm.2023.01.005)
Supplement: Multimedia component 1 [file mmc1.pdf]

Appendix A. Supplementary Data

Date	New	Cumulative	Date	New	Cumulative
7/18/2020	1	1	12/3/2020	2	25
7/22/2020	1	2	12/9/2020	1	26
7/23/2020	1	3	12/11/2020	1	27
7/24/2020	1	4	12/16/2020	1	28
7/27/2020	1	5	12/17/2020	1	29
7/29/2020	1	6	12/28/2020	3	32
8/4/2020	1	7	12/30/2020	2	34
9/1/2020	1	8	12/31/2020	1	35
10/12/2020	1	9	1/4/2021	3	39
10/18/2020	1	10	1/5/2021	1	40
10/19/2020	1	11	1/12/2021	1	41
10/23/2020	1	12	1/13/2021	2	43
10/26/2020	1	13	1/14/2021	2	45
10/31/2020	1	14	1/16/2021	2	47
11/12/2020	1	15	1/19/2021	1	48
11/16/2020	1	16	1/21/2021	1	49
11/25/2020	1	18	1/25/2021	1	50
11/29/2020	1	19	1/28/2021	2	52
11/30/2020	1	20	2/3/2021	1	53
12/1/2020	1	21	2/5/2021	1	54
12/2/2020	2	23	2/10/2021	1	55

Table A.3: UTCVM COVID-19 data, new and cumulative cases.

Appendix B. Disease-Free equilibrium and the calculation of R_0

The disease-free equilibrium (DFE) for our model is given by:

$$X^* = (S_v^*, S_p^*, 0, 0, 0, 0, 0, 0, 0, 0) = \left(\frac{\Gamma_1}{d}, \frac{\Gamma_2}{d}, 0, 0, 0, 0, 0, 0, 0, 0\right)$$

To investigate the stability of the disease-free equilibrium, we use the Next Generation Matrix method developed in [48] to calculate the basic reproductive number $\mathcal{R}_0$. We consider $E_v, E_p, A_v, A_p, I_v, I_p$. as infected compartments and S_v, S_p, Q, R as noninfected compartments. Note that Q is an isolated compartment. First, we find the rates of new infections for all infected compartments, and call this $\mathcal{F}$.

$$\begin{bmatrix} v_1 \left[\frac{\beta_{vv} v_1 (A_v + bI_v)}{v_1 N_v} + \frac{\beta_{vp}(1+c)p_1(A_p+bI_p)}{v_1 N_v + p_1 N_p}\right] S_v + (1-v_1)\left[\frac{\beta_{vp}(1-p_1)(A_p+bI_p)}{(1-p_1)N_p + (1-v_1)N_v}\right] S_v \\ (1-p_1)\left[\frac{\beta_{pp}(1-p_1)(A_p+bI_p)}{(1-p_1)N_p+(1-v_1)N_v}\right] S_p + p_1 \frac{\beta_{vp} v_1 (A_v+bI_v)}{v_1 N_v} S_p \\ 0 \\ 0 \\ 0 \\ 0 \end{bmatrix}$$

Then, we find the rates of transition for the infected compartments and call this $\mathcal{V}$.

$$\begin{bmatrix} \alpha E_v + dE_v \\ \alpha E_p + dE_p \\ -\alpha\sigma E_v + \gamma_A A_v + \gamma A_v + dA_v \\ -\alpha\sigma E_p + \gamma_A A_p + dA_p \\ -\alpha(1-\sigma)E_v + \gamma_I I_v + dI_v \\ -\alpha(1-\sigma)E_p + (\gamma_I + \gamma_R) I_p + dI_p \end{bmatrix}$$

We calculate the Jacobian matrix from new infections, $\mathbf{F}$.

$$\mathbf{F}(\mathbf{X}^*) = \begin{bmatrix} 0 & 0 & f_1 & f_2 & f_3 & f_4 \\ 0 & 0 & f_5 & f_6 & f_7 & f_8 \\ 0 & 0 & 0 & 0 & 0 & 0 \\ 0 & 0 & 0 & 0 & 0 & 0 \\ 0 & 0 & 0 & 0 & 0 & 0 \\ 0 & 0 & 0 & 0 & 0 & 0 \end{bmatrix}$$

with $f_1 = v_1\beta_{vv}, f_2 = \frac{v_1 S_v^* \beta_{vp}(1+c)p_1}{v_1 S_v^* + p_1 S_p^*} + \frac{(1-v_1)S_v^*\beta_{vp}(1-p_1)}{(1-p_1)S_p^* + (1-v_1)S_v^*}, f_3 = v_1 b\beta_{vv}$, $f_4 = \frac{v_1 S_v^* \beta_{vp}(1+c)p_1}{v_1 S_v^* + p_1 S_p^*} + \frac{(1-v_1)S_v^*\beta_{vp}(1-p_1)}{(1-p_1)S_p^* + (1-v_1)S_v^*}, f_5 = \frac{\beta_{vp} p_1 S_p^*}{S_v^*}, f_6 = \frac{(1-p_1)\beta_{pp}(1-p_1)S_p^*}{(1-p_1)S_p^* + (1-v_1)S_v^*}$, $f_7 = \frac{p_1\beta_{vp} b S_p^*}{S_v^*}, f_8 = \frac{(1-p_1)\beta_{pp}(1-p_1)bS_p^*}{(1-p_1)S_p^* + (1-v_1)S_v^*}$. We calculate the Jacobian matrix of $\mathcal{V}$, called $\mathbf{V}$.

$$\mathbf{V}(\mathbf{X}^*) = \begin{bmatrix} v_1 & 0 & 0 & 0 & 0 & 0 \\ 0 & v_1 & 0 & 0 & 0 & 0 \\ v_2 & 0 & v_3 & 0 & 0 & 0 \\ 0 & v_2 & 0 & v_4 & 0 & 0 \\ v_5 & 0 & 0 & 0 & v_6 & 0 \\ 0 & v_5 & 0 & 0 & 0 & v_7 \end{bmatrix}$$

with $v_1 = \alpha + d, v_2 = -\alpha\sigma, v_3 = \gamma_A + \gamma + d, v_4 = \gamma_A + d, v_5 = -\alpha(1-\sigma)$, $v_6 = \gamma_I + d, v_7 = \gamma_I + \gamma_R + d$. We need to calculate the inverse of $\mathbf{V}$ to find

the Next Generation Matrix.

$$\mathbf{V^{-1}} = \begin{bmatrix} \frac{1}{v_1} & 0 & 0 & 0 & 0 & 0 \\ 0 & \frac{1}{v_1} & 0 & 0 & 0 & 0 \\ -\frac{v_2}{v_1 v_3} & 0 & \frac{1}{v_3} & 0 & 0 & 0 \\ 0 & -\frac{v_2}{v_1 v_4} & 0 & \frac{1}{v_4} & 0 & 0 \\ -\frac{v_5}{v_1 v_6} & 0 & 0 & 0 & \frac{1}{v_6} & 0 \\ 0 & -\frac{v_5}{v_1 v_7} & 0 & 0 & 0 & \frac{1}{v_7} \end{bmatrix}$$

The product $\mathbf{FV^{-1}}$ at the DFE gives us the Next Generation Matrix and the spectral radius of this matrix gives the basic reproductive number $\mathcal{R}_0$ which is a threshold value for the DFE to be locally stable:

$$\mathbf{FV^{-1}}(\mathbf{X}^*) = \begin{bmatrix} n_1 & n_2 & n_3 & n_4 & n_5 & n_6 \\ n_7 & n_8 & n_9 & n_{10} & n_{11} & n_{12} \\ 0 & 0 & 0 & 0 & 0 & 0 \\ 0 & 0 & 0 & 0 & 0 & 0 \\ 0 & 0 & 0 & 0 & 0 & 0 \\ 0 & 0 & 0 & 0 & 0 & 0 \end{bmatrix}$$

with $n_1 = -\frac{v_6 v_2 f_1 + f_3 v_5 v_3}{v_1 v_3 v_6}, n_2 = -\frac{v_7 v_2 f_2 + v_4 v_5 f_4}{v_1 v_4 v_7}, n_3 = \frac{f_1}{v_3}, n_4 = \frac{f_2}{v_4}, n_5 = \frac{f_3}{v_6}$, $n_6 = \frac{f_4}{v_7}, n_7 = -\frac{v_6 v_2 f_5 + f_7 v_5 v_3}{v_1 v_3 v_6}, n_8 = -\frac{v_7 v_2 f_6 + v_4 v_5 f_8}{v_1 v_4 v_7}, n_9 = \frac{f_5}{v_3}, n_{10} = \frac{f_6}{v_4}, n_{11} = \frac{f_7}{v_6}$, $n_{12} = \frac{f_8}{v_7}$. Thus, we obtain

$$\mathcal{R}_0 = \frac{n_1 + n_8 + \sqrt{n_1^2 + 4n_2 n_7 - 2n_1 n_8 + n_8^2}}{2}$$

If this value is less than one, then the DFE is locally stable, otherwise the DFE is unstable.

Note that in calculating R_0 numerically, we used $\beta_{vp} = 0.03, d = 2.3562 \cdot 10^{-5}$,

$\Gamma_1 = 14.6488 \cdot (300/470000), \Gamma_2 = 14.6488$ [49], $\beta_{vp} = 0.03$, in addition to the parameters in Table 2.

References

[1] T. Carvalho, F. Krammer, A. Iwasaki, The first 12 months of COVID-19: a timeline of immunological insights, Nature Reviews Immunology 21 (2021) 245–256. doi:10.1038/s41577-021-00522-1.

[2] J. R. Fauver, M. E. Petrone, E. B. Hodcroft, K. Shioda, H. Y. Ehrlich, A. G. Watts, C. B. Vogels, A. F. Brito, T. Alpert, A. Muyombwe, et al., Coast-to-coast spread of SARS-CoV-2 during the early epidemic in the United States, Cell 181 (5) (2020) 990–996. doi:10.1016/j.cell.2020.04.021.

[3] Z. Zhuang, S. Zhao, Q. Lin, P. Cao, Y. Lou, L. Yang, S. Yang, D. He, L. Xiao, Preliminary estimates of the reproduction number of the coronavirus disease (COVID-19) outbreak in Republic of Korea and Italy by 5 March 2020, International Journal of Infectious Diseases 95 (2020) 308–310. doi:10.1016/j.ijid.2020.04.044.

[4] M. Renardy, M. Eisenberg, D. Kirschner, Predicting the second wave of COVID-19 in Washtenaw County, MI, Journal of Theoretical Biology 507 (2020) 110461. doi:10.1016/j.jtbi.2020.110461.

[5] C. Xu, Y. Dong, X. Yu, H. Wang, L. Tsamlag, S. Zhang, R. Chang, Z. Wang, Y. Yu, R. Long, Y. Wang, G. Xu, T. Shen, S. Wang, X. Zhang, H. Wang, Y. Cai, Estimation of reproduction numbers of COVID-19 in
